# Better together: building protein oligomers naturally and by design

**DOI:** 10.1042/BST20190283

**Published:** 2019-12-05

**Authors:** Rebecca E.A. Gwyther, D. Dafydd Jones, Harley L. Worthy

**Affiliations:** 1 Molecular Biosciences, School of Biosciences, Cardiff University, Cardiff, U.K.; 2 School of Biosciences, University of Exeter, Exeter, U.K.

**Keywords:** expanded genetic code, oligomerization, protein complexes, protein engineering, structural biology, synthetic biology

## Abstract

Protein oligomers are more common in nature than monomers, with dimers being the most prevalent final structural state observed in known structures. From a biological perspective, this makes sense as it conserves vital molecular resources that may be wasted simply by generating larger single polypeptide units, and allows new features such as cooperativity to emerge. Taking inspiration from nature, protein designers and engineers are now building artificial oligomeric complexes using a variety of approaches to generate new and useful supramolecular protein structures. Oligomerisation is thus offering a new approach to sample structure and function space not accessible through simply tinkering with monomeric proteins.

## Introduction

**Figure 1.**
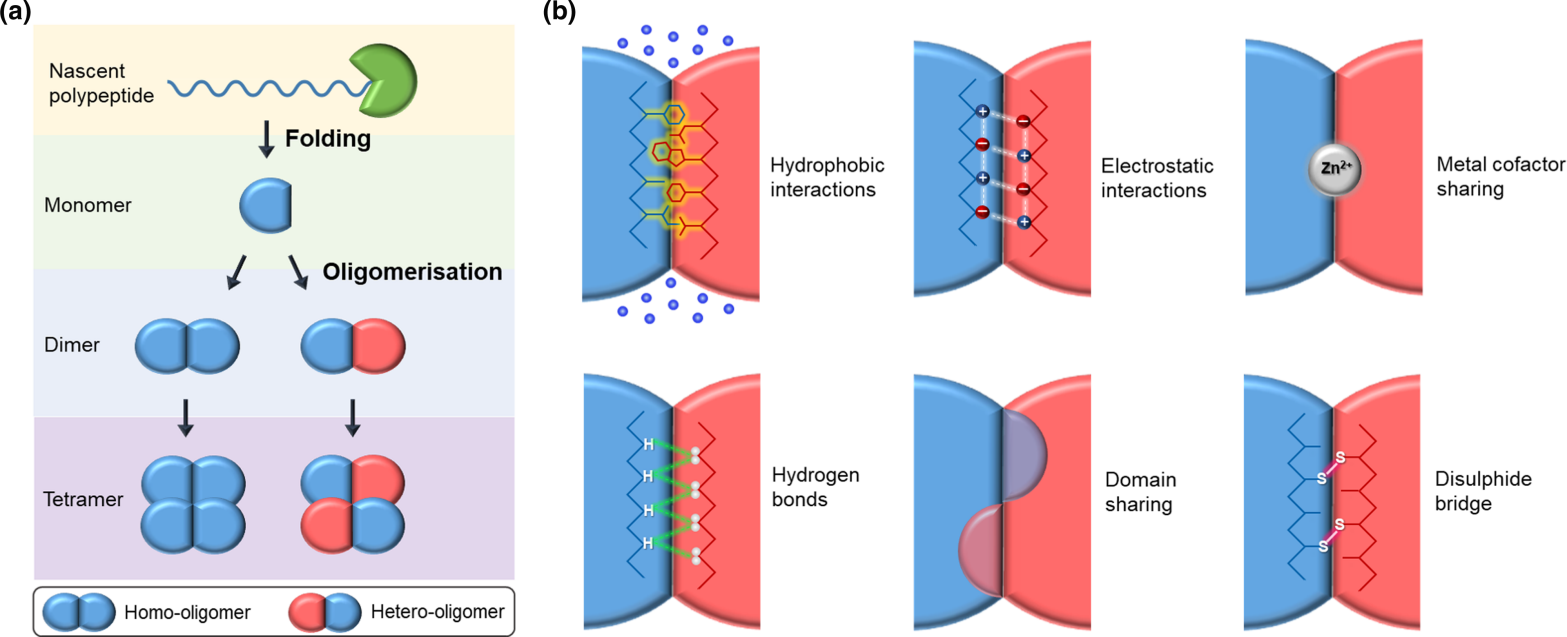
Oligomerisation of proteins. (**a**) Oligomer formation. Nascent polypeptides enter the endoplasmic reticulum and undergo folding to form their monomeric structure. Monomers can then partake in a process of oligomerisation to form dimers, tetramers and other higher order oligomers. Association of the same monomer forms homo-oligomers, whilst interaction with a different monomer will form hetero-oligomers. (**b**) Mechanisms of interaction at the oligomeric interface to form a mutually compatible interface. Non-polar residues can mediate hydrophobic interactions, whilst polar residues can facilitate hydrogen bonds and, to a lesser extent, electrostatic interaction. Less common approaches utilise domain and metal cofactor sharing to bring monomers together. Disulfide bridges are the main if relatively rare covalent mechanism mediating oligomerisation, and this is often exploited artificially to oligomerise two polypeptides.

The vast expanse of protein sequence space has been the driving force behind molecular evolution, with the quaternary structure adding another level of diversity so further expanding the variation observed. Individual polypeptide chains amalgamate to produce a macromolecular complex through a process of oligomerisation (Figure 1a). The molecular coupling of individual subunits leads to new structural and functional properties beyond those available in a simple monomeric system so offering evolutionary benefits, especially when starting from a limited monomer repertoire. This is apparent from the dominance of oligomers over monomers as a final structural form for proteins; with symmetrical homodimers representing the most commonly observed form in the Protein Databank [1,2].

The fundamental basis behind protein oligomerisation is the network of intricate interactions holding the individual units together. Protein oligomers are normally formed and stabilised by a variety of mechanisms, the most common of which is the formation of a mutually compatible non-covalent interface (Figure 1b). The interface is stabilised by hydrophobic interactions, H-bonds and electrostatic attraction [3,4]. Other less common mechanisms include domain swapping (e.g. β-strand exchange, as seen in dimeric cytokines and some cytochromes [5]), binding of shared metal cofactors (e.g. insulin [3,6]) and formation of inter-protein disulfide bridges to connect monomers, with linking of the light and heavy chains in antibodies being an excellent example [1–3,7,8]. Crucially, the total sum of interaction between monomers dictate its strength, which is varied advantageously to provide temporal and spatial regulation over protein complex interactions. Examples of this are evident in large complex structures like the cytoskeleton and collagen [2,9], and multienzyme complexes such as RNA polymerase and pyruvate dehydrogenase [3], to small functional dimers such as cell receptors and transcription factors [3,9,10]. However, looking beyond the interactions that drive oligomerisation, there are several reasons behind the triumph of the protein oligomer; and these are something synthetic biologists need to consider when designing novel proteins and genomes.

## Fundamentals of oligomeric success

Given the abundance of oligomeric proteins, a key question is what makes oligomeric proteins so evolutionarily successful and why are the majority symmetrical? [3,9] Numerous people have attempted to answer this including Klotz [11] and Monod et al. [12] in the 1960s, and more recently by both Goodsell and Olson [3], and Ali and Imperiali [9], who reviewed the topic at the turn of the century. General conclusions included that larger proteins were seen as preferential to smaller proteins, because they are more resistant to denaturation and degradation through reduced solvent to surface area exposure [3,4,11,12]. There is also the advantage of having multiple active sites allowing for cooperative functionality. For example, large multienzyme complexes, such as RNA polymerase have an increased turnover rate compared with the subunits acting independently [3]. Whilst these are benefits that may have been achieved through larger single proteins, nature has favoured the use of smaller subunits to generate the same effect.

Multiple proteins subunits offer a safety net for translation errors; by creating a large protein complex of monomers, subunits with an error can be quickly discarded without great strain on the cells’ resources [3]. In prokaryotes, ∼25% of proteins of 500 amino acids or more contain an amino acid substitution and ^1^/_7_ of proteins are released from the ribosome before the full-length protein is created [13,14]. This means that proteins greater than 2000 amino acids are rarely fully translated and when they are translated the protein contains at least one error [13,14]. There is also an increased risk of misfolding with longer polypeptide chains having a more complex folding energy landscape and as such may require chaperone proteins [13,14]. A final advantage of utilising multiple subunits is increased coding efficiency at the DNA level. For example, a protein with 1000 amino acids could be coded by a single gene 3 kb long (not including regulatory DNA) or could be made up of four identical subunits 250 amino acids in length, requiring a gene of only 750 bp. Not only does this save genetic space and space within the cell, energy is conserved through more efficient replication and transcription of the subunits [2,3,15]. This, combined with monomeric interactions, are key factors to consider when designing novel oligomeric proteins.

## Designing artificial oligomers

Designing and producing artificial self-assembling protein complexes is currently of great interest to protein engineering, and a variety of inventive techniques [5,16,17] have been used to mediate this, including fusion proteins/domains [18] (e.g. Nanohedra [19] and protein nanobuilding blocks [20,21]), split proteins/domains (e.g. Spycatcher [22] and split luciferase domains [23]), helix-helix interactions [24–26], metal ion bridging [27–29], cofactor bridging [30], and disulfide bridging [7] — to name a few [31–34].

The primary reason for developing artificial oligomers is to explore the new structure and function space not currently present in nature and to use these new designs to try and solve technological, medical and scientific problems [16,35,36]. For example, using domain insertion of split luciferase domains has been used to create Rho GTPase biosensors, where one half of the luciferase is genetically incorporated into the GTPase and the second part of luciferase is attached to potential ligands [23]. If the ligand binds to the GTPase then the two fragments of luciferase combine to form a functionally active bioluminescent protein [23]. Using the same split-domain technique, the Spycatcher–Spytag system was developed and used for localisation and assisted purification of proteins of interest [22,37], Meanwhile, Bailey et al. [38] pursued a different a strategy: metal-coordination chemistry to direct assembly of protein oligomers. This resulted in the creation of protein nanotubes and lattices with variable diameters [28,39], whilst Song and Tezcan [27] used the strategy to engineer rudimentary β-lactamase activity into a cytochrome-based scaffold.

Most of the techniques mentioned above rely on using structural information gleaned from the protein databank based on oligomers found in nature [28,33,36]. This information is then used to engineer proteins of interest to conform to a set of rules to generate dimerisation interfaces [10,25,40]. These interfaces multimerise because they form hydrophobic patches that associate to escape solvent, hydrogen bonding networks where many weak interactions create a stable interface [2], or the halves of an interface co-ordinate a metal ion [11,12] or cofactors like heme [2,9,30]. A potential downside to these methodologies is the requirement for an extensive design process and significant engineering of proteins to incorporate oligomerisation into normally monomeric systems. Relying solely on symmetry can also limit the shapes and lattices that can be explored [41].

A potential alternative to the empirical-based dimer design is disulfide cross-linking via engineered cysteine residues. As mentioned above, engineering cysteines into proteins to form inter-protein cross-links is an approach used by nature and popular amongst researchers, requiring very little modification of the target protein (usually only a single residue). Functional covalent dimers of azurin were created using this method, mutating residue Asn42 to cysteine and allowing the formation of a disulfide linkage between two monomers [42]. However, the dimers showed a decrease in electron transfer, which was attributed to reduced flexibility from the short disulfide linker [42]. The short side chain of cysteine (–CH_2_–SH) will make many interaction interfaces sterically impossible and when generating hetero-complexes or beyond dimers, off-pathway disulfide bonds will generate a mix of oligomeric states. Other risks accompanying this method include the increased likelihood of misfolded proteins from the newly mutated cysteines forming incorrect disulfide bridges with existing cysteine residues [43], and the unsuitability for *in vivo* work, due to the reducing environment of most living cells [43].

## Use of non-canonical amino acids

**Figure 2.**
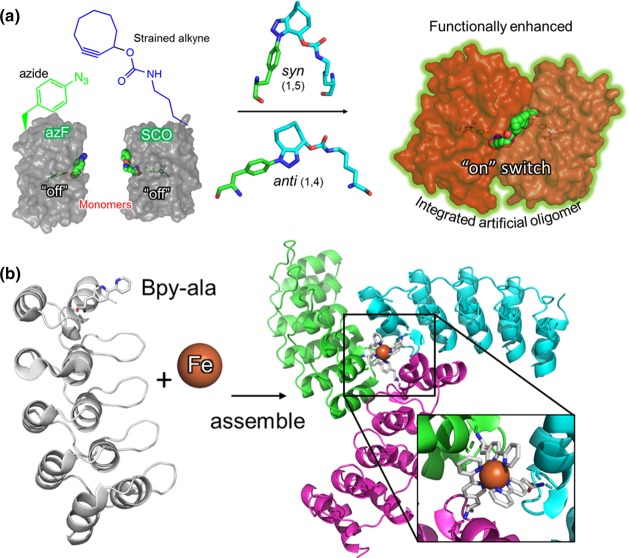
Oligomerisation of proteins via designed ncAA incorporation. (**a**) Dimerisation of sfGFP^ncAA^ monomers. A scheme depicting the strain promoted azide-alkyne cycloaddition between two non-fluorescent monomers containing azF and SCO (left), forming either ‘*syn*’ or ‘*anti*’ triazole linkages (centre) to generate a fluorescent dimer (dimer). In the case above, the original monomeric proteins are largely inactive that are switched on when dimerised through linkage via residue 148 (green spheres, PDB 5NHN). Figure is copied with permission from Worthy et al. [62] under a creative commons license. (**b**) Scheme showing the oligomerisation of a synthetic ankyrin repeat protein (grey), containing the metal-chelating ncAA 2,2′-bipyridin-5yl-alanine (Bpy-ala, shown as sticks). Briefly, upon addition of iron ions (orange sphere, Fe), three protein monomers assemble to form a spiral pattern. The iron atom forms the nucleus of the trimer co-ordinated by three Bpy-ala residues. (PDB 5EIL [20]).

Through the use of non-canonical amino acids (ncAAs) coupled with codon reprogramming new chemistries have been introduced into proteins by design which overcome the site specificity issues [44–47]. An example of these bioorthogonal chemistries is strain promoted azide-alkyne cycloaddition (SPAAC) [48]. Incorporation of ncAAs and the SPAAC reaction has been extensively reviewed and studied elsewhere [47,49–55]. In theory, incorporating an azide moiety (e.g. p-azido-l-phenylalanine, AzF) into one protein and an alkyne (e.g. s-cyclooctyne-l-lysine, SCO) into another (Figure 2a), it is possible to create defined covalent protein dimers in defined orientations. Other examples of generating protein oligomers using ncAAs include the synthesis of antibody dimers [56], ubiquitin [57] dimers, metal-chelating homotrimers [29] (Figure 2b), and multifunctional enzymatic complexes [58,59], which use a variety different ncAAs: p-acetylphenylalanine [56], azidohomoalanine [57,59], (2,2′-bipyridin-5yl)alanine [29], and other aliphatic and aromatic alkyne derivatives [60,61].

The Jones group has successfully demonstrated the use of genetically encoded SPAAC by linking monomers of fluorescent proteins together to create functionally enhanced dimers of super-folder green fluorescent protein (sfGFP) (Figure 2a) [62]. The fluorescence mechanism and properties of sfGFP and other *Aequorea victoria* derived FPs is well defined [63–66] with the central active component being the solvent-shielded chromophore, buried within the β-barrel structure. Composed of residues 65 (variable in variants of GFP and Thr in sfGFP), Tyr66 and Gly67, the chromophore can exist in two protonation states: the less populated CRO A, featuring a neutral protonated phenol group of Tyr66, or the more fluorescent and highly populated CRO B with a charged phenolate; switching between these two states gives rise to its characteristic spectral properties [64,65]. Residue His148 plays a crucial role in the deprotonation of Tyr66 [63]. Mutation of H148 to a ncAA results in the breakage of this key H-bond causing the CRO A chromophore to predominate [51,54]. The formation of sfGFP homodimers using SPAAC compatible residues at 148 not only reverses this protonation state so switching on CRO B, but enhances brightness over threefold above wild type sfGFP — indicative of functional synergy [62]. Comparison of the 400 nm : 485 nm excitation peaks would thus allow a ratiometric estimation of the CRO A monomer to CRO B dimer population. The study of the structures arising from these artificial protein dimers suggests that the improved fluorescence is due to the formation of extended hydrogen bonding networks between both chromophores. This work paves the way for not just linking monomeric proteins together but shows how generating intimate interactions can lead to new emergent properties.

## Using protein dimerisation to monitor protein–protein interactions

The archetypal technique for monitoring protein–protein interactions (PPIs) is fluorescent biosensors, which transduce real-time ligand-binding events into a measurable fluorescence signal [67]. These proximity-based biosensors have numerous advantages over alternative strategies, including their selectivity and sensitivity in spectral analysis, temporal and spatial resolution in biomolecular imaging and relative low cost [68,69]. However, these properties vary inherently between different subtypes of the fluorescent biosensor, bringing selective advantages and disadvantages to each application.

Fluorescence resonance energy transfer (FRET) [70,71], utilises the overlapping emission and excitation spectra of two different fluorophores to stimulate a change in fluorescence when their proximity is <10 nm [72]. This becomes a useful experimental tool when fusing the fluorophores to two potential interaction partners/domains, as the fluorescence output should correlate with their proximity, and thus interaction. Limitations to this, however, include the low signal-to-noise ratio (SNR) from background autofluorescence and the sensitivity of fluorescent proteins (FPs) to changes in their microenvironment [73]. Plus, the most abundant oligomerisation event, homo-dimerisation cannot be easily monitored. Biomolecular fluorescent complementation overcomes the background autofluorescence of FRET by physically splitting the FPs and attaching the two halves to putative interacting proteins, restoring emission only when an interaction occurs [74,75]. Nevertheless, limitations here are often temporally linked: slow off-rates between the split fragments prevent time-dependent studies, delays in fluorescent readouts arise from protein folding and chromophore maturation and false-positives arising from non-specific self-assembly [76]. The final biosensing approach involves engineering single FPs to respond to analytes directly by incorporating receptor elements into FP design [77–80]. This approach effectively increases the temporal perception but is hampered by the complex design process; with a prerequisite for precise structural knowledge and conformational change modelling to ensure correct protein folding upon analyte binding [81].

In an attempt to expand this repertoire of proximity-based biosensors, dimerisation-dependent FP (ddFP) biosensors have become a new focus for the scientific community [82–84]. This strategy typically involves the formation of a fluorescent heterodimer from two non-fluorescent counterparts: a quenched monomer and a monomer lacking a chromophore [84]. Attaching these FPs to separate interacting proteins brings the complex together to form a FP dimer and so enhancing the fluorescence output and thus the SNR. However, considering the aforementioned developments in mutations centred on residue 148 of sfGFP mutants [51,62], it is exciting to note the potential homodimers may have used as a novel suite of ddFP biosensors. Not only will these be easier to engineer than heterodimers, homodimers are likely to be more stable to fluctuations in the microenvironment. This is because monomers should respond in a near-enough identical manner; whereas heterodimers will experience inherent variation and as such, unpredictably skew fluorescent measurements. It also allows ratiometric sensing which removes concentration-dependent measurement issues. Additionally, it opens up the ability to monitor homo-oligomeric complexes as only one engineered FP is needed to achieve a response. Overall, oligomerisation is shown to be a fantastic tool for monitoring PPIs, and future developments in this area will likely have a widespread impact across biological research.

## Conclusion

Focusing on converting perceived ‘hard-to-use' oligomeric proteins to monomers limits the available function space open to the protein designer and engineer. Taking inspiration from nature and its concept of building up complex supramolecular structures from simple components (protein monomers), we can now envisage assembling the complex protein–based structure. Approaches available to expedite such ‘bottom-up' assembly are expanding all the time taking in classical protein engineering to *in silico* design to the use of new chemistry types. Such potential is already being shown with the simple peptide-based system (reviewed by Beesley and Woolfson [85]). We should also consider incorporating different molecular types, even including abiological materials such as the exciting nano-carbon system [86]. So sometimes, it is better for proteins to be together.

**Perspectives**

- Oligomeric proteins complexes are the most common structural form of proteins found in nature.
- Taking inspiration from nature new artificial protein oligomeric systems are being constructed to sample new functional and structural features not accessible in monomeric proteins
- Major steps have been taken in constructing artificial protein oligomers but more is to come in terms of function, complexity, applications and even inclusion of non-biological components

## Abbreviation

ddFP: dimerisation-dependent FP
FRET: Fluorescence resonance energy transfer
ncAAs: non-canonical amino acids
PPIs: protein–protein interactions
sfGFP: super-folder green fluorescent protein
SNR: signal-to-noise ratio
SPAAC: strain promoted azide-alkyne cycloaddition
